# Licochalcone A Prevents Platelet Activation and Thrombus Formation through the Inhibition of PLCγ2-PKC, Akt, and MAPK Pathways

**DOI:** 10.3390/ijms18071500

**Published:** 2017-07-12

**Authors:** Li-Ming Lien, Kuan-Hung Lin, Li-Ting Huang, Mei-Fang Tseng, Hou-Chang Chiu, Ray-Jade Chen, Wan-Jung Lu

**Affiliations:** 1 School of Medicine, College of Medicine, Taipei Medical University, Taipei 110, Taiwan; M002177@ms.skh.org.tw; 2 Department of Neurology, Shin Kong Wu Ho Su Memorial Hospital, Taipei 111, Taiwan; M001012@ms.skh.org.tw; 3 Department of Pharmacology and Graduate Institute of Medical Sciences, Taipei Medical University, Taipei 110, Taiwan; d102092002@tmu.edu.tw; 4 Central Laboratory, Shin Kong Wu Ho Su Memorial Hospital, Taipei 111, Taiwan; 5 Department of Medical Research and Division of General Surgery, Department of Surgery, Taipei Medical University Hospital, Taipei 110, Taiwan; tiffany4441@gmail.com (L.-T.H.); viola0928@hotmail.com (M.-F.T.); 6 College of Medicine, Fu-Jen Catholic University, Taipei 242, Taiwan; 7 Graduate Institute of Metabolism and Obesity Sciences, College of Public Health and Nutrition, Taipei Medical University, Taipei 110, Taiwan

**Keywords:** Licochalcone A, middle cerebral artery occlusion, platelet activation, PLCγ2–PKC, thrombus formation

## Abstract

Platelet activation is involved in cardiovascular diseases, such as atherosclerosis and ischemic stroke. Licochalcone A (LA), an active ingredient of licorice, exhibits multiple biological activities such as anti-oxidation and anti-inflammation. However, its role in platelet activation remains unclear. Therefore, the study investigated the antiplatelet mechanism of LA. Our data revealed that LA (2–10 μM) concentration dependently inhibited platelet aggregation induced by collagen, but not thrombin and U46619. LA markedly attenuated collagen-stimulated ATP release, P-selectin secretion, calcium mobilization, and GPIIbIIIa activation, but did not interfere with the collagen binding to platelets. Moreover, LA significantly reduced the activation of PLCγ2, PKC, Akt and MAPKs. Thus, LA attenuates platelet activation, possibly by inhibiting collagen receptor downstream signaling but not by blocking the collagen receptors. In addition, LA prevented adenosine diphosphate (ADP)-induced acute pulmonary thrombosis, fluorescein sodium-induced platelet thrombus formation, and middle cerebral artery occlusion/reperfusion-induced brain injury in mice, but did not affect normal hemostasis. This study demonstrated that LA effectively reduced platelet activation and thrombus formation, in part, through the inhibition of PLCγ2–PKC, Akt, and MAPK pathways, without the side effect of bleeding. These findings also indicate that LA may provide a safe and alternative therapeutic approach for preventing thromboembolic disorders such as stroke.

## 1. Introduction

Platelet activation is involved in normal hemostasis and in pathological processes such as atherosclerosis and stroke [1,2]. When blood vessels are injured, exposed extracellular matrix proteins (e.g., collagen and von Willebrand factor) activate platelets and further recruit additional platelets from the bloodstream. These platelets form a firm platelet plug at the injury site to stop blood loss; however, under pathological conditions, the platelets are prone to uncontrolled activation and aggregation and may cause vessel occlusion.

Collagen signaling is mediated through the interaction of collagen and its receptor glycoprotein VI (GPVI) and integrin α2β1 [3], which are located on the plasma membrane of platelets; these receptors transmit the activation signals, including those of phospholipase Cγ2 (PLCγ2) and protein kinase C (PKC) activation, and mediate platelet granule release and calcium mobilization [3], all of which finally lead to glycoprotein IIbIIIa (GPIIbIIIa) activation and subsequent platelet aggregation. These processes are crucial for platelet activation and thrombus formation [4]. Moreover, GPVI serves as a promising pharmacological target for the effective and safe treatment of thrombotic and possibly inflammatory diseases [4]. Aspirin and clopidogrel are commonly used to prevent stroke, but account for only a 20% reduction in all recurrent stroke events [5]. Thus, targeting collagen signaling may provide an alternative therapeutic approach to mitigate the recurrence of secondary stroke.

Licochalcone A (LA), a natural chalcone derived from the roots and rhizomes of *Glycyrrhiza* spp., exhibits multiple biological activities such as antibacterial, antioxidant, anti-inflammatory, antimalarial, antiviral, and antitumor effects [6,7,8,9,10,11]. LA inhibits lipopolysaccharide (LPS)-induced reactive oxygen species (ROS) production and cytokine release in the RAW 264.7 mouse macrophage cell line [12]. LA also alleviates LPS-induced acute lung and kidney injury through NF-κB and p38/ERK mitogen-activated protein kinase (MAPK) signaling and attenuates pertussis toxin-induced autoimmune encephalomyelitis by reducing the production of tumor necrosis factor-α and interferon-γ in vivo [12,13,14]. In addition, LA induces tumor cell cycle arrest, apoptosis, and autophagy in a various cancer cell lines [10,11]. These observations reveals that LA protects against several pathological processes.

Although, recently, LA was also reported to reduce rabbit and rat platelet activation through the inhibition of cyclooxygenase-1 (COX-1) activity [15,16], its role in platelet activation and thrombosis remains unclear. In the present study, our preliminary data revealed that LA significantly inhibited collagen-induced platelet aggregation through PLCγ2–PKC pathway, suggesting that, in addition to inhibiting COX-1 activity, LA may attenuate platelet activation through other mechanisms. Therefore, we further systemically investigated the mechanism of LA in platelet activation and for the first time determined whether LA has antithrombotic effect in in vivo studies.

## 2. Results

### 2.1. Licochalcone A (LA) Inhibited Collagen-Induced Platelet Aggregation

**Figure 1:**
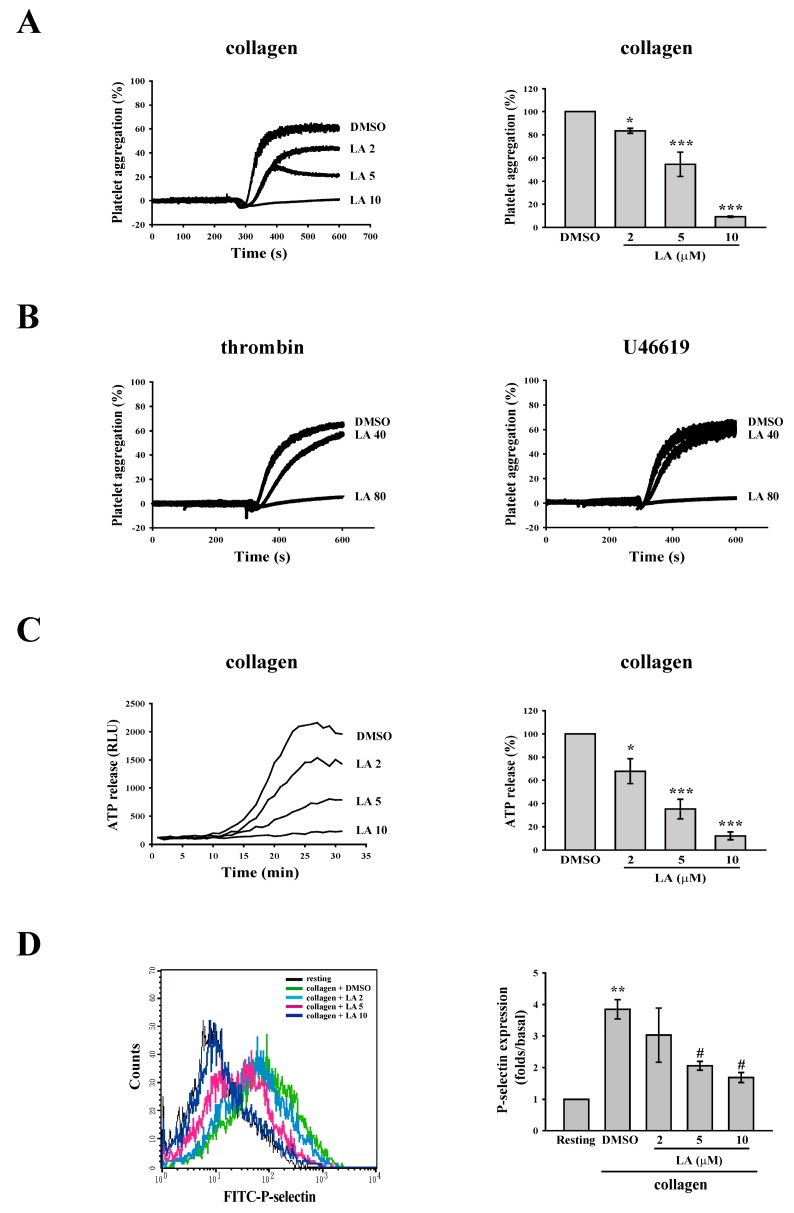
Effects of Licochalcone A (LA) on the collagen-induced platelet aggregation, ATP release, and P-selectin secretion. (**A**,**B**) Washed platelets (3.6 × 10^8^ cells/mL) were preincubated with DMSO (solvent control) and LA (2–80 µM), and were then stimulated using collagen (1 µg/mL), thrombin (0.01 U/mL), or U46619 (1 µM) to trigger platelet aggregation, as measured by a transmission aggregometer; (**C**) The effect of LA on collagen-induced ATP release was characterized by the detection of chemiluminescent emission from the luciferin–luciferase reaction, which was continually recorded using a microplate reader; (**D**) The effect of LA on collagen-induced P-selectin secretion was detected using FITC–P-selectin antibody. The fluorescence was immediately detected through flow cytometry. The profiles (**B**) are representative examples of five similar experiments. Data (**A**,**C**) are presented as the mean ± SEM. (**A**, *n* = 5; **C**, *n* = 3). * *p <* 0.05 and *** *p <* 0.001, compared with the DMSO group; Data (**D**) are presented as the mean ± SEM. (*n* = 3). ** *p <* 0.01, compared with the resting group; ^#^ *p <* 0.05, compared with the collagen (positive) group.

As shown in Figure 1A, LA (2–10 µM) was used to determine its effect on the platelet aggregation induced by collagen (1 µg/mL). The data indicated that LA (at concentrations of 2, 5 and 10 µM) inhibited collagen-induced platelet aggregation by 16.6%, 45.3% and 90.8%, respectively. The IC_50_ was approximately 5.6 µM. In addition, only at a higher concentration of 80 µM did LA affect thrombin (0.01 U/mL)- or U46619 (1 µM)-mediated platelet aggregation (Figure 1B). These results are consistent with that Okuda-Tanino et al. reported [15] and indicate that LA is more sensitive to the inhibition of collagen-mediated platelet activation. Accordingly, in the following experiments, we mainly evaluated the mechanism of LA at concentrations of 2–10 µM in collagen-mediated platelet activation events.

### 2.2. LA Inhibited Collagen-Mediated ATP Release and P-Selectin Secretion

Platelet granule release plays a crucial role in the amplification of platelet activation and aggregation. Here, two experiments on ATP release and P-selectin secretion, which were assessed by the microplate reader and through flow cytometry, respectively, were performed to determine whether LA interferes with the granule release induced by collagen. As shown in Figure 1C, luciferase/luciferin was used to detect ATP. The data revealed that collagen markedly induced ATP release, which was reversed by LA (2–10 µM) in a concentration-dependent manner. Moreover, LA (5 and 10 µM) significantly reduced collagen-induced P-selectin secretion (Figure 1D), as determined by the intensity of fluorescein isothiocyanate (FITC)–P-selectin. These findings suggest that LA inhibits collagen-mediated platelet activation through the blockade of granule release.

### 2.3. LA Inhibited Collagen-Mediated Calcium Mobilization and GPIIbIIIa Activation without Interfering with Collagen Receptors

Calcium signaling is the common platelet activation signaling pathway. Receptor-stimulated PLC catalyzes the hydrolysis of phosphatidylinositol biphosphate to release inositol trisphosphate and diacyglycerol, which activate calcium mobilization and PKC, respectively [17]. The elevation of intracellular Ca^2+^ contributes to several events of platelet activation, such as shape change, granule release, and GPIIbIIIa activaiton [18].

**Figure 2:**
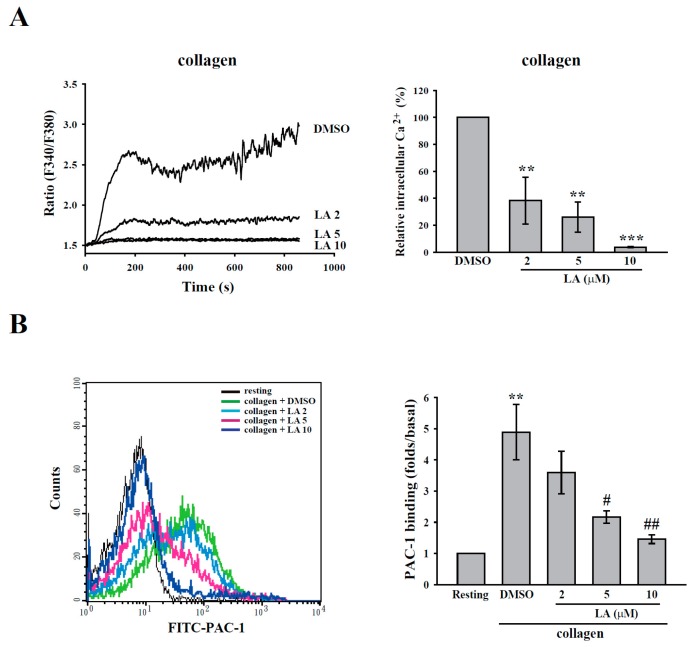

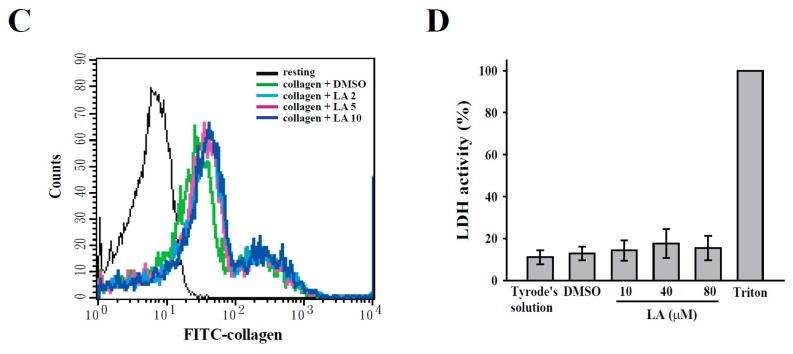
Effects of LA on calcium mobilization, GPIIbIIIa activation, collagen receptors and lactate dehydrogenase (LDH) release. Washed platelets were preincubated with DMSO and LA (2–10 µM), and were then stimulated using 1 µg/mL collagen (**A**,**B**) or FITC–collagen (**C**). (**A**) The ratio of fura-2 fluorescence (F340/F380) was used to determine calcium mobilization, as measured by a Hitachi F4500 fluorescence spectrophotometer; (**B**,**C**) GPIIbIIIa activation and the competition with collagen were determined using the FITC–PAC-1 antibody and FITC–collagen, respectively. The fluorescence intensity was measured through flow cytometry; (**D**) The platelets were preincubated with Tyrode’s solution, DMSO (solvent control) or various concentrations of LA (10–80 µM) for 10 min at 37 °C, and the supernatant was collected to measure LDH release by the LDH assay kit. LDH activity was expressed as the % of total enzyme activity, which was measured in platelets lysed with 0.5% Triton X-100. Data (**A**) are presented as the means ± SEM (*n* = 3). ** *p <* 0.01 and *** *p <* 0.001, compared with the DMSO group; Data (**B**) are presented as the means ± SEM (*n* = 3). ** *p <* 0.01, compared with the resting group ^#^ *p <* 0.05 and ^##^ *p <* 0.01, compared with the collagen (positive) group. Profiles (**C**,**D**) are representative examples of three similar experiments.

As shown in Figure 2A, fura-2 was used to measure the change in calcium level according to the ratio of F340/F380, which is directly correlated to the amount of intracellular calcium. The data revealed that LA (2–10 µM) markedly inhibited calcium mobilization, as detected using F-4500 Fluorescence Spectrophotometer. Moreover, the FITC–PAC-1 antibody was used to demonstrate that LA (5–10 µM) markedly inhibited GPIIbIIIa activation (Figure 2B), a final step in platelet aggregation, as detected through flow cytometry. These findings indicate that LA blocks calcium mobilization and subsequent GPIIbIIIa activation, thereby inhibiting platelet aggregation. In addition, FITC–collagen was used to determine whether LA directly blocks collagen receptors, leading to the inhibition of its downstream signaling. As shown in Figure 2C, the data obtained from flow cytometry revealed that FITC–collagen markedly binds to platelets. Moreover, pretreated LA did not interfere with the binding of FITC–collagen to platelets. This finding suggests that LA inhibits collagen-induced platelet activation, possibly by inhibiting collagen receptor downstream signaling but not by blocking the collagen receptors. In addition, LA (10–80 µM) did not exhibit cytotoxic effects on platelets, as detected by the LDH assay (Figure 2D), indicating that LA-mediated the inhibition of platelet activation is not due to the cytotoxicity.

### 2.4. LA Inhibited Collagen-Mediated Platelet Activation Signaling

**Figure 3:**
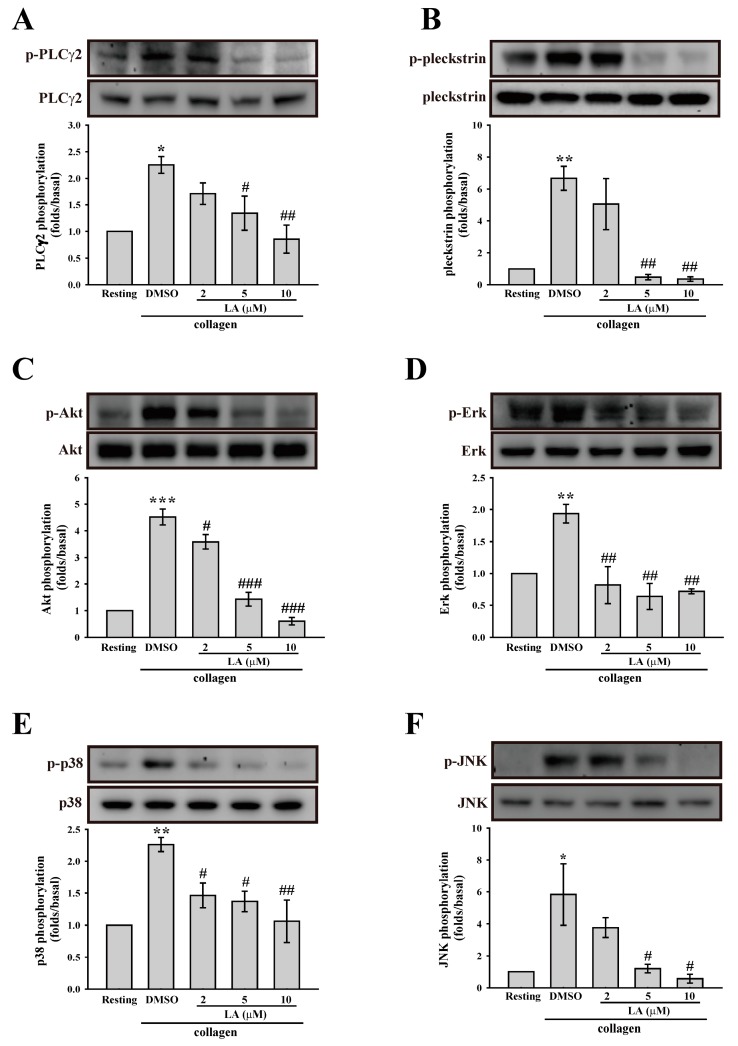
Involvement of LA in the activation of (**A**) PLCγ2; (**B**) PKC; (**C**) Akt; (**D**) Erk; (**E**) p38 mitogen-activated protein kinase (MAPK) and (**F**) JNK. Washed platelets (3.6 × 10^8^ cells/mL) were preincubated with DMSO and LA (2–10 µM), and collagen (1 µg/mL) was then added to trigger platelet activation. Cells were then collected, and subcellular extracts were analyzed through Western blotting. Specific antibodies were used to detect the phosphorylation of PLCγ2, the PKC substrate pleckstrin, Akt, Erk, p38 MAPK, and JNK. Data (**A**–**F**) are presented as the mean ± SEM (*n* = 3). * *p* < 0.05, ** *p* < 0.01 and *** *p* < 0.001, compared with the resting group; ^#^ *p* < 0.05, ^##^ *p* < 0.01 and ^###^ *p* < 0.001, compared with the collagen (positive) group.

Collagen mediates platelet activation, mainly by clustering the collagen receptor, GPVI. Therefore, in the present study, we determined the effect of LA on GPVI downstream signaling. As shown in Figure 3A, LA significantly inhibited the collagen-induced PLCγ2 phosphorylation. In addition, the activation of PKC, the downstream of PLCγ2, was also determined. In platelets, the phosphorylation of the major PKC substrate p47 protein (approximately 47 kDa), also known as pleckstrin, has been used to measure PKC activation [19]. Moreover, our data also revealed that the PKC inhibitor Ro318220 (2 µM) markedly inhibited collagen-mediated the phosphorylation of pleckstrin, indicating that this phosphorylation is PKC-dependent (Figure S1). As shown in Figure 3B, LA also inhibited PKC activation (pleckstrin phosphorylation), indicating that LA inhibits platelet activation, in part, through the inhibition of the PLCγ2–PKC pathway. In addition to the common PLCγ2–PKC pathway, the activation of Akt and MAPKs, including Erk, p38 MAPK, and JNK, is involved in collagen-mediated platelet aggregation [20,21]. Hence, we also determined the role of LA in these pathways. As shown in Figure 3C–F, collagen could stimulate the phosphorylation of Akt, p38, JNK, and Erk, and this effect was reversed by LA (2–10 µM). These findings indicate that LA attenuated GPVI downstream signaling, thereby blocking the platelet aggregation induced by collagen.

### 2.5. LA Alleviated ADP-Induced Pulmonary Thrombosis and Fluorescein Sodium-Induced Platelet Thrombus Formation in the Mesenteric Microvessels of Mice

**Figure 4:**
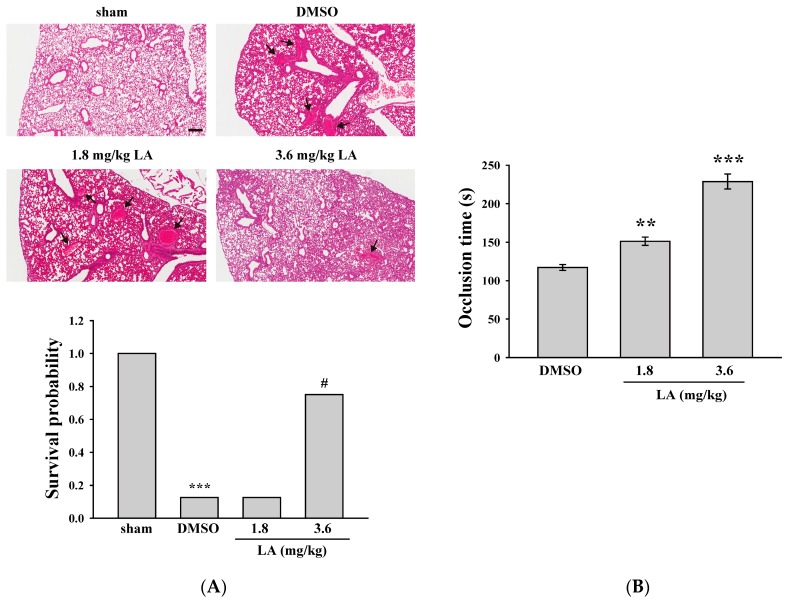
Effects of LA on pulmonary thrombosis and fluorescein sodium-induced platelet thrombus formation in the mesenteric microvessels of mice. (**A**) Mice (male, 5–6 weeks old) were intraperitoneally administered with DMSO (solvent control) or LA (1.8 and 3.6 mg/kg) for 30 min. ADP (1.4 g/kg) was injected in the tail vein to induce acute pulmonary thrombosis. The survival rate (bottom panel) was determined at 1 and 24 h after ADP administration, and pulmonary thrombosis (top panel, arrows) was observed by staining lung tissue sections with hematoxylin-eosin. Scale bar: 200 μm. The survival rate was evaluated using the Kaplan–Meier survival method (*n* = 8). *** *p <* 0.001, compared with the sham-operated group. ^#^ *p <* 0.05, compared with the DMSO group; (**B**) Mice received an intravenous bolus of DMSO or LA (1.8 and 3.6 mg/kg), and the mesenteric venules were irradiated to induce microthrombus formation. Data are presented as the mean ± SEM (*n* = 8). ** *p <* 0.01 and *** *p <* 0.001, compared with the DMSO group.

In the following experiments, we used several animal models to determine the effect of LA on thrombus formation. In the lung thrombosis model of mice, ADP (1.4 g/kg) was used to induce acute pulmonary thrombosis. As shown in Figure 4A (top panel), the lungs of the mice were stained with hematoxylin-eosin. The data revealed that the DMSO group exhibited severe pulmonary thrombosis (arrows), whereas a higher dose of LA (3.6 mg/kg) exerted marked protective effects. In addition, the survival rate of mice was determined at 1 h after ADP was administered (Figure 4A, bottom panel). The DMSO (solvent control) group had a survival rate of only 12.5% (1/8). Only the higher dose of LA (3.6 mg/kg) effectively increased the survival rate to 75% (6/8, *p <* 0.05).

Fluorescein sodium was used in another model of platelet thrombus formation in mesenteric microvessels; this model was exposed to UV irradiation, which damaged endothelium and subsequently caused vascular occlusion. The occlusion time was recorded using a real-time monitor. As shown in Figure 4B, the data revealed that the DMSO group had an occlusion time of approximately 117.2 s. Compared with the DMSO group, LA (1.8 and 3.6 mg/kg) treatment dose-dependently prolonged the occlusion time by 34.0 and 111.5 s (both *p <* 0.01, *n* = 8), respectively. The findings obtained from these two animal models indicate that LA exerts anti-thrombotic effects.

### 2.6. LA Protected against Middle Cerebral Artery Occlusion/Reperfusion-Induced Brain Injury without Affecting Normal Hemostasis

**Figure 5:**
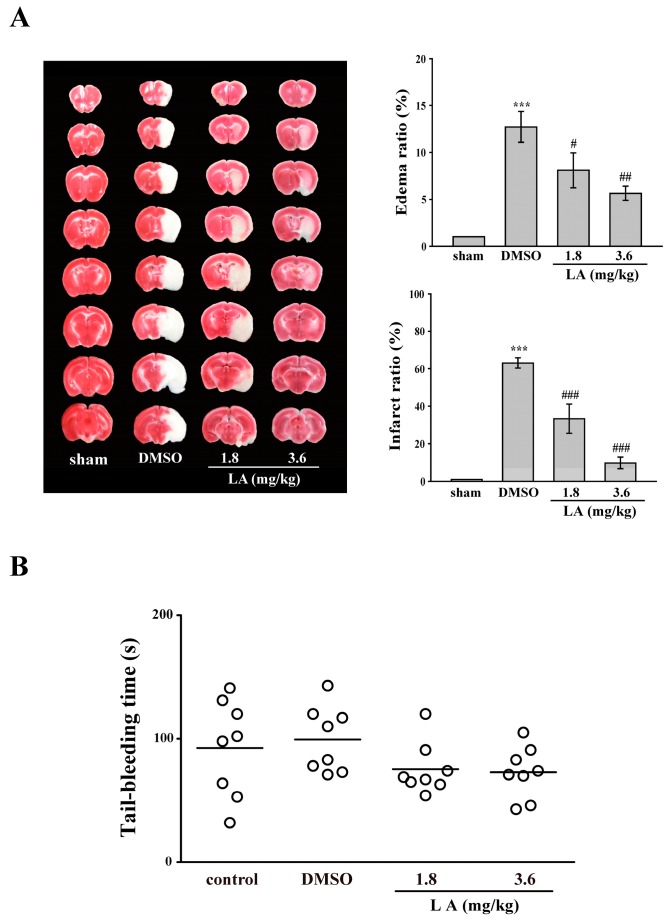
Influence of LA on middle cerebral artery occlusion (MCAO)/reperfusion-induced brain injury and tail bleeding time in mice. Mice (male, 5–6 weeks old) were intraperitoneally administrated with DMSO (solvent control) or LA (1.8 and 3.6 mg/kg) for 30 min. (**A**) Mice were subjected to MCAO for 30 min followed by 24-h reperfusion. Immediately after sacrifice, coronal sections were cut and stained using 2,3,5-triphenyltetrazolium chloride; white areas indicate infarction, and red areas indicate normal tissues (left panel). Edema and infarct ratios (right panel) were calculated through image analysis and are reported as a ratio of the non-ischemic hemisphere. Infarct ratio was corrected for edema. Data are presented as the mean ± SEM. (*n* = 8). *** *p* < 0.001, compared with the sham-operated group; ^#^ *p* < 0.05, ^##^ *p* < 0.01 and ^###^ *p* < 0.001, compared with the DMSO group; (**B**) Bleeding was induced by severing the tail at 3 mm from the tail tip, and the bleeding tail stump was immersed in saline. Subsequently, the bleeding time was continually recorded until no sign of bleeding was observed for at least 10 s. Each point in the scatter plots graph represents a mouse (*n* = 8). The bars represent the median bleeding time of each group.

In clinical settings, anti-platelet drugs have been used to prevent secondary stroke. Moreover, previous studies on experimental stroke have revealed the crucial role of platelet activation, in addition to inflammatory responses [22,23,24,25]. Hence, we investigated whether LA has a protective effect on middle cerebral artery occlusion (MCAO)-induced brain injury. As shown in Figure 5A, the data described a marked edema (12.6%) and infarct size (61.6%) in the DMSO group. However, the LA treated groups showed a dose-dependent reduction in edema (1.8 mg/kg, 6.0%; 3.6 mg/kg, 4.7%) and infarct size (1.8 mg/kg, 27.7%; 3.6 mg/kg, 8.4%), compared with the DMSO group. This finding indicates that LA protects against MCAO-induced brain injury, at least in part, through the inhibition of platelet activation.

As previously described, LA can reduce thrombus formation and protect against stroke-mediated brain injury. However, in clinical settings, the side effect of bleeding caused by antiplatelet drugs remains a challenge. Hence, we performed an experiment on tail-bleeding time to evaluate whether LA interferes with normal hemostasis. As shown in Figure 5B, the data indicated that the average tail-bleeding time was approximately 92.6 and 99.4 s in the control (saline) and DMSO groups, respectively. There is no significant difference between these two groups (*p >* 0.05, *n* = 8). In addition, the LA-treated groups did not prolong the tail-bleeding time, compared with the DMSO group, indicating that LA is a safer antithrombotic agent.

## 3. Discussion

Medicinal herbs, including those used in traditional medicine are becoming increasingly popular and important in Western countries [26,27]. In China and other countries, traditional medicine has long used medicinal herbs to treat or relieve the symptoms of many human diseases [26,27]. Moreover, the active ingredients of herbs or natural products are among the most crucial resources for developing new lead compounds and scaffolds for treating human diseases [26,27]. Therefore, research on herbs or natural products is a key field for developing new drugs for treating human diseases.

LA is an active ingredient derived from the roots and rhizomes of *Glycyrrhiza* spp.; it possesses multiple biological activities, such as anti-inflammation, antioxidation, antibacteria, and antitumor effects [7,9,10,11]. Although, LA was recently reported to block rabbit and rat platelet aggregation through the inhibition of COX-1 activity [15,16], the role of LA on platelet activation and thrombosis remains unclear. Therefore, we further systemically investigated its antiplatelet mechanism and for the first time determined whether LA prevents thrombus formation in in vivo studies. Our data reveal that LA is more sensitive to the inhibition of collagen-induced platelet aggregation in human platelets, as well as that which Okuda-Tanino et al. reported [15]. Moreover, LA also inhibits collagen-mediated several activation events, such as granule release, calcium mobilization, and GPIIbIIIa activation. In addition, we further determined the possible antiplatelet mechanisms of LA. Several GPVI-mediated signaling pathways were examined in this study. LA could inhibit the conventional PLCγ2–PKC pathway as well as Akt and MAPK pathways. GPVI-mediated Akt activation in platelets is majorly dependent on PI3K and, in part, on Gi protein stimulation by secreted ADP, indicating that PI3K/Akt is the direct downstream target of GPVI [20]. Moreover, the pharmacological inhibition of PI3K markedly inhibit GPVI-mediated platelet aggregation, secretion, and intracellular calcium mobilization [20]. Similar to the pharmacological inhibitors, the gene deletion of Akt1 impairs collagen-induced platelet activation [28]. These findings indicates that LA inhibits collagen-induced platelet activation, in part, through the inhibition of Akt activation, followed by reducing ADP release.

MAPKs, including Erk, p38 MAPK, and JNK, are also involved in collagen-induced platelet activation [21]. P2X_1_-mediated Erk2 activation is essential for platelet secretion and aggregation, which is induced by a low dose of collagen (≤1 mg/mL) [21]. Moreover, the Erk upstream MEK1/2 inhibitor prolongs the occlusion time of the arteriolar and venular thrombosis in mice [21]; p38 MAPK activates cytosolic phospholipase A2, leading to subsequent increased thromboxane A_2_ formation in collagen-stimulated platelets [21]. Likewise, p38 inhibitors impair platelet aggregation in response to low and medium doses of collagen. Furthermore, p38 MAPK is involved in thrombus formation, as evidenced in *p38*^+^^/−^ mice in a model of ferric chloride-induced carotid artery occlusion [21]; the deletion of JNK1 also impairs platelet aggregation and granule release by a low dose of collagen and thrombus formation in an in vivo model of thrombosis induced by photochemical injury to cecum vessels [29]. These observations suggest that LA could reduce collagen-induced platelet activation, including granule release, GPIIbIIIa activation, and platelet aggregation, partly through the inhibition of Erk, p38 MAPK, and JNK activation. In addition, previous studies have demonstrated that LA inhibits COX-1 activity in different species [15,16]. Our data also showed that LA effectively reduced AA-induced human platelet aggregation (Figure S2). This result indirectly implies that LA may has the inhibitory effect on COX-1 activity in human platelets. Collectively, in addition to inhibiting COX-1 activity that Okuda-Tanino et al. and Suo et al. reported [15,16], LA-mediated the inhibition of the PLCγ2-PKC, Akt, and MAPK pathways also contributes to the suppression of platelet activation induced by collagen. Furthermore, the inhibitory effect of LA on platelet activation does not occur through interference with the collagen binding to GPVI, as evidenced by the FITC-collagen binding assay (Figure 2C). Thus, LA may inhibit GPVI downstream signaling, but does act as an antagonist to GPVI.

LA markedly attenuated thrombus formation in two animal models of ADP-induced pulmonary thrombosis and fluorescein sodium-induced platelet thrombus formation in the mesenteric microvessels of mice. Moreover, LA did not affect normal hemostasis. These findings suggest that LA is a relatively safe antithrombotic agent. In our study, our data also showed that LA could inhibit mouse platelet aggregation (Figure S3), which, in part, supports that LA has protective effects in ADP-induced pulmonary thrombosis of mice. However, the influence of LA on ADP-induced platelet aggregation is controversial. LA reportedly inhibited ADP-induced platelet aggregation in rat, but not rabbit, platelets [15,16]. Thus, whether this diversity is attributed to the different species remains to be further clarified. In addition, LA protected against MCAO-induced brain injury. In this ischemia/reperfusion model, several risk factors, including neutrophil infiltration, ROS production, and platelet activation, are involved in the processes of brain injury [22,23,24,25]. Actually, previous studies have demonstrated that LA has a potent anti-inflammatory activity in various models, including acute kidney and lung injury, allergic airway inflammation, and endotoxin shock [12,14,30,31]. LA also exhibits anti-oxidant activity; it protects against tert-butyl hydroperoxide-induced oxidative stress by scavenging ROS, and inhibits LPS-induced ROS production, lipid peroxidation, and nitric oxide in RAW 264.7 cells [7,32]. In addition to the antiplatelet effect, these reported biological activities of LA may also contribute to a protective benefit for thrombosis and ischemic stroke.

## 4. Materials and Methods

### 4.1. Materials

Licochalcone A (LA, ≥95%) was purchased from Cayman Chemical (Ann Arbor, MI, USA). Collagen, thrombin, and U46619 were purchased from Chrono-Log (Havertown, PA, USA). FITC-conjugated anti-P-selectin and PAC-1 antibodies were purchased from Biolegend (San Diego, CA, USA). FITC-conjugated collagen, phorbol-12, 13-dibutyrate (PDBu), luciferase/luciferin, fluorescein sodium, and 2,3,5-triphenyltetrazolium chloride (TTC) were purchased from Sigma (St. Louis, MO, USA). Fura 2-AM was purchased from Molecular Probe (Eugene, OR, USA). Anti-phospho PLCγ2 (Tyr^759^), anti-PLCγ2, anti-phospho-(Ser) PKC substrate, anti-phospho-p38 MAPK (Ser^180^/Tyr^182^), anti-phospho-p44/42 MAPK (ERK1/2; Thr^202^/Tyr^204^), anti-c-Jun N-terminal kinase (JNK), and anti-phospho-Akt (Ser^473^) polyclonal antibodies and anti-p38 MAPK, anti-p44/42 MAPK, anti-phospho JNK (Thr^183^/Tyr^185^), and anti-Akt monoclonal antibodies were purchased from Cell Signaling (Beverly, MA, USA). The pleckstrin (p47) antibody was purchased from GeneTex (Irvine, CA, USA). The Hybond-P polyvinylidene difluoride membrane, an enhanced chemiluminescence (ECL) Western blotting detection reagent and analysis system, horseradish peroxidase (HRP)-conjugated donkey antirabbit IgG, and sheep antimouse IgG were purchased from Amersham (Buckinghamshire, UK). LA was dissolved in dimethyl sulfoxide (DMSO) and stored at 4 °C until use.

### 4.2. Platelet Aggregation

This study was approved by the Institutional Review Board of Shin Kong Wu Ho-Su Memorial Hospital (Approval No. 20161205R, 9 February 2017) and conformed to the directives of the Helsinki Declaration. All volunteers provided written informed consent before any procedure of experiments. Blood was collected from healthy volunteers who were free from medication during the past 2 weeks, and prepared to human platelet suspensions, as previously described [33,34]. Blood was mixed with an acid‑citrate-dextrose solution (9:1, *v*/*v*). Following centrifugation, the supernatant platelet-rich plasma (PRP) was collected. Then PRP was supplemented with prostaglandin E1 (0.5 µM) and heparin (6.4 IU/mL) before the second centrifugation. Washed platelets were suspended in Tyrode’s solution containing bovine serum albumin (BSA) (3.5 mg/mL). The final concentration of Ca^2+^ in Tyrode’s solution was 1 mM.

A turbidimetric method was applied to measure platelet aggregation [33,34] by using a Lumi-Aggregometer (Payton, Scarborough, ON, Canada). Platelet suspensions (3.6 × 10^8^ cells/mL) were preincubated with various concentrations of LA (2–80 µM) or an isovolumetric solvent control (0.1% DMSO, final concentration) for 3 min before agonists were added. The reaction was allowed to proceed for 6 min.

### 4.3. ATP Release Measured Using a Microplate Reader

Platelet suspensions (3.6 × 10^8^ cells/mL) were preincubated with luciferase/luciferin and various concentrations of LA or an isovolumetric solvent control (0.1% DMSO, final concentration) for 3 min before collagen was added. The reaction was allowed to proceed for 30 min and the luminescence was continually recorded every minute using a Synergy H1 microplate reader (BioTek, VT, USA).

### 4.4. P-Selectin Secretion and GPIIbIIIa Activation

This method was previously described by Yacoub et al. [35]. Platelet suspensions (3 × 10^8^ platelets/mL) were preincubated with LA (2–10 µM) or 0.1% DMSO for 3 min, and subsequently, collagen (1 µg/mL) was added for 15 min in glass cuvettes at 37 °C. After the reactions, the platelet suspensions were fixed and stained with FITC–P-selectin or FITC–PAC-1 antibody for 30 min. After centrifugation and washing, platelets were re-suspensioned with 1 mL phosphate-buffered saline and all samples were immediately measured in a Becton Dickinson flow cytometer (FACScan Syst., San Jose, CA, USA). The number of events was stopped at 10,000 counts. All of the experiments were performed at least three times to ensure reliability.

### 4.5. Calcium Mobilization

The washed platelets was incubated with Fura 2-AM (5 µM) for 30 min, which allowed Fura 2 to cross the cell membrane and to be trapped within platelets. After centrifugation and washing, platelets were suspended with Tyrode’s solution. The final concentration of Ca^2+^ in Tyrode’s solution was 1 mM. The real-time change of relative intracellular Ca^2+^ ion ([Ca^2+^]i) concentration was recorded by a fluorescence spectrophotometer (Hitachi F4500, Tokyo, Japan) with excitation wavelengths of 340 and 380 nm and an emission wavelength of 500 nm [36].

### 4.6. Determination of Lactate Dehydrogenase (LDH)

LDH release was analyzed using a CytoTox 96 non-radioactive cytotoxicity assay kit from Promega (Madison, WI, USA). Washed platelets (3.6 × 10^8^ cells/mL) were pre-incubated with LA (10, 40, and 80 µM) or a solvent control (0.1% DMSO, final concentration) for 10 min at 37 °C. After centrifugation, an aliquot of supernatant was collected to measure the levels of LDH according to manufacturer’s protocol (Promega). The levels of LDH were measured at the wavelength of 490 nm using a Synergy H1 microplate reader (BioTek, VT, USA). LDH activity was expressed as the % of total enzyme activity, which was measured in platelets lysed with 0.5% Triton X-100.

### 4.7. Immunoblotting Study

Washed platelets (3 × 10^8^ cells/mL) were treated with 1 µg/mL collagen to trigger platelet activation for the indicated times in the absence or presence of LA (2–10 µM). After the reaction, all samples was collected and immediately lysed in 200 µL of a lysis buffer for 1 h. The lysates were centrifuged at 5000× *g* for 5 min. The amounts of 80 µg of protein lysates were loaded into each well, separated on a 12% SDS-PAGE, and electrotransferred to PVDF membranes through semidry transfer (Bio-Rad, Hercules, CA, USA). PVDF membranes were blocked with 5% BSA in TBST (Tris-base 10 mM, NaCl 100 mM and Tween 20 0.01%) for 1 h at room temperature, and then probed with various specific primary antibodies (diluted at 1:1000 in TBST), followed by incubation with the HRP-linked antimouse IgG or antirabbit IgG (diluted at 1:3000 in TBST) for 1 h. An enhanced chemiluminescence system was used to develop the immunoreactive bands on the membranes. Then the optical density of each band was measured by a videodensitometry software (Bio-Profil; Biolight Windows Application V2000.01, Vilber Lourmat, France); the density of each band was normalized to the corresponding total protein band.

### 4.8. Competitive Binding Assay of Collagen Receptors through Flow Cytometry

For the flow cytometry, platelet suspensions (1 × 10^6^ platelets/mL) were pre-incubated with LA (2–10 µM) or 0.1% DMSO for 3 min, and subsequently, FITC–collagen (1 µg/mL) was added for 15 min in glass cuvettes at 37 °C. After the reaction, a final volume of 1 mL was used for an immediate analysis through flow cytometry (Becton Dickinson, FACScan Syst., San Jose, CA, USA).

### 4.9. Animals

ICR and C57BL/6 mice (20–25 g, male, 5–6 weeks old) were obtained from BioLasco (Taipei, Taiwan). All procedures were approved by the Affidavit of Approval of Animal Use Protocol-Shin Kong Wu Ho-Su Memorial Hospital (Approval No. Most1060005, 22 December 2016) and were in accordance with the Guide for the Care and Use of Laboratory Animals (8th edition, 2011). LA (5 and 10 µM) effectively platelet activation in vitro, these two concentrations were chosen and calculated accordingly into mouse doses 1.8 and 3.6 mg/kg, respectively [37].

### 4.10. ADP-Induced Acute Pulmonary Thrombosis in Mice

This experiment was performed as previously described [38]. Mice were injected with ADP (1.4 g/kg) at the tail vein to induce acute pulmonary thrombosis. The survival rate of the mice in each group was determined at 1 h after injection. Mice that survived the challenge were euthanized in a CO_2_ chamber. The lungs were excised and fixed with 4% formalin, and paraffin-embedded sections of the lungs were stained with hematoxylin-eosin. The stained sections were observed and photographed using ScanScope CS (Leica Biosystems, Wetzlar, Germany). The mice were divided into four groups: (1) sham-operated; (2) DMSO (solvent control); (3) LA-treated (1.8 mg/kg, intraperitoneal (i.p.)) and (4) the LA-treated (3.6 mg/kg, i.p.). All treatments were administered 30 min before ADP administration for all the groups except for the sham-operated group.

### 4.11. Fluorescein Sodium-Induced Platelet Thrombus Formation in Mesenteric Microvessels of Mice

Thrombus formation was assessed as previously described [39]. Mice were anesthetized using a mixture containing 75% air and 3% isoflurane maintained in 25% oxygen, and the external jugular vein was cannulated with a polyethylene 10 (PE-10) tube for intravenously administering the dye and drugs. Venules (30–40 mM) were selected for irradiation at wavelengths of <520 nm to produce a microthrombus. Either 1.8 or 3.6 mg/kg of LA was administered at 10 min after the administration of sodium fluorescein (15 mg/kg), and the time required to occlude the microvessel through thrombus formation (occlusion time) was recorded.

### 4.12. Middle Cerebral Artery Occlusion/Reperfusion-Induced Brain Injury in Mice

In this experiment of stroke, the intraluminal suture method was used, as described previously [40]. In brief, after mice were anesthetized with a mixture of 75% air and 25% oxygen containing 3% isoflurane, the right common carotid artery was exposed. Then, the right middle cerebral artery (MCA) was occluded by inserting a 6–0 monofilament nylon suture coated with silicon from the external to the internal carotid artery until no longer advanceable. After the closure of the operative site, the mice were allowed to recover from anesthesia. During another brief anesthesia period, the suture was gently withdrawn to restore blood supply after 30 min of MCAO. All groups of mice were euthanized through decapitation after 24 h reperfusion. The brains were cut into 1-mM coronal slices and stained with 2% 2,3,5-triphenyltetrazolium chloride. The infarct areas were calculated using a computerized image analyzer (Image-Pro Plus, Rockville, MD, USA) and then compiled to obtain the infarct volume (in cubic millimeters) per brain. Infarct volumes were expressed as a percentage of the contralateral hemisphere volume by using the formula (area of the intact contralateral (left) hemisphere-area of the intact region of the ipsilateral (right) hemisphere) to compensate for edema formation in the ipsilateral hemisphere. Four groups were designed as follows: (1) sham-operated; (2) DMSO (solvent control); (3) LA-treated (1.8 mg/kg, i.p.) and (4) LA-treated (3.6 mg/kg, i.p.). All treatments were administered 30 min prior to the onset of MCAO in all the groups except for the sham-operated group.

### 4.13. Tail Bleeding Time

Mice were anesthetized with a mixture containing 75% air and 3% isoflurane maintained in 25% oxygen and were intraperitoneally administered with saline (control), DMSO (solvent control), or LA (1.8 or 3.6 mg/kg) for 30 min. Immediately, bleeding was induced by severing the tail at 3 mm from the tail tip, and the bleeding tail stump was immersed in saline. Subsequently, the bleeding time was continually recorded until no sign of bleeding was observed for at least 10 s.

### 4.14. Data Analysis

The experimental results are expressed as the mean ± SEM. and are accompanied by the number of observations (*n*). Values of n refer to the number of experiments, each of which was conducted with different blood donors. All experimental results were assessed using analysis of variance (ANOVA). Significant differences were investigated using the Newman–Keuls method. Survival rates were calculated using the Kaplan–Meier method, and the groups were compared using the log rank test. Results with *p* < 0.05 were considered statistically significant.

## 5. Conclusions

**Figure 6:**
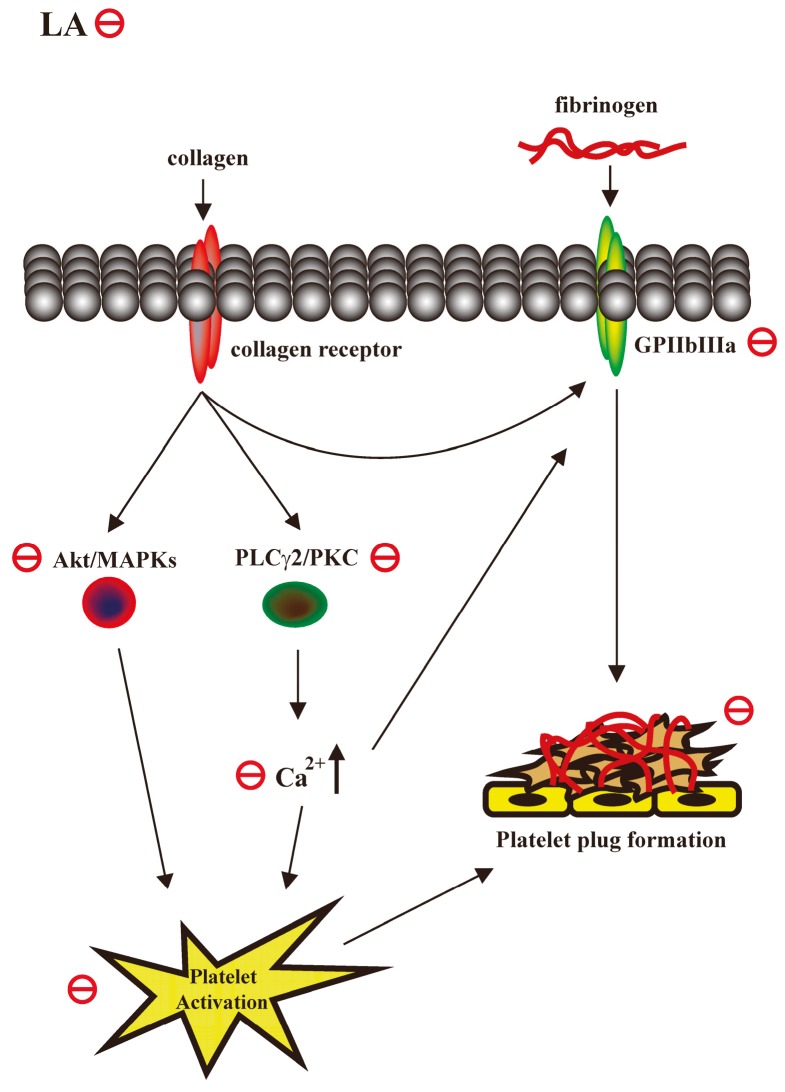
Hypothetical scheme of the involvement of LA in human platelet activation. LA may inhibit PLCγ2–PKC, Akt and MAPK activation and subsequently attenuate granule release and GPIIbIIIa activation, thereby blocking platelet aggregation and thrombus formation. Arrows indicate positive regulation; red circles indicate inhibition.

The major findings of this study revealed that LA prevents platelet activation and thrombus formation partly through the inhibition of PLCγ2–PKC, Akt, and MAPK pathways (Figure 6), without affecting normal hemostasis. These findings indicate the potential of LA as a safe and effective therapy for preventing thromboembolic disorders such as secondary stroke. In addition, LA may serve as a leading compound for the development of novel antithrombotic drugs.

## Supplementary Materials

Supplementary materials can be found at www.mdpi.com/1422-0067/18/7/1500/s1.

## Abbreviations

GPIIbIIIa	Glycoprotein IIbIIIa
GPVI	Glycoprotein VI
LA	Licochalcone A
MAPK	Mitogen-activated protein kinase
MCAO	Middle cerebral artery occlusion
PKC	Protein kinase C
PLCγ2	Phospholipase Cγ2
ROS	Reactive oxygen species
