# Supplementary material for: Licochalcone A Prevents Platelet Activation and Thrombus Formation through the Inhibition of PLCγ2-PKC, Akt, and MAPK Pathways
Source: Int J Mol Sci. 2017 Jul 12;18(7):1500. doi: 10.3390/ijms18071500 (PMC5535990; doi:10.3390/ijms18071500)
Supplement: Supplementary file 1 [file ijms-18-01500-s001.pdf]

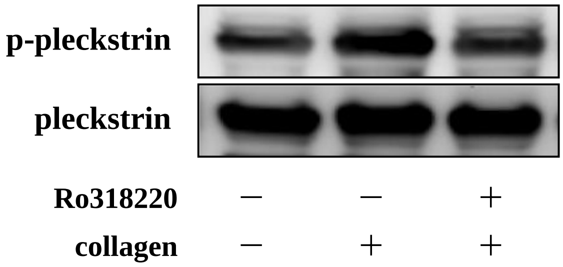

Figure S1. Effects of Ro318220 on the phosphorylation of pleckstrin. Washed human platelets were treated with collagen (1 μg/mL) to trigger platelet activation in the absence or presence of Ro318220 (2 μM). Profiles are representative of 3 similar experiments.

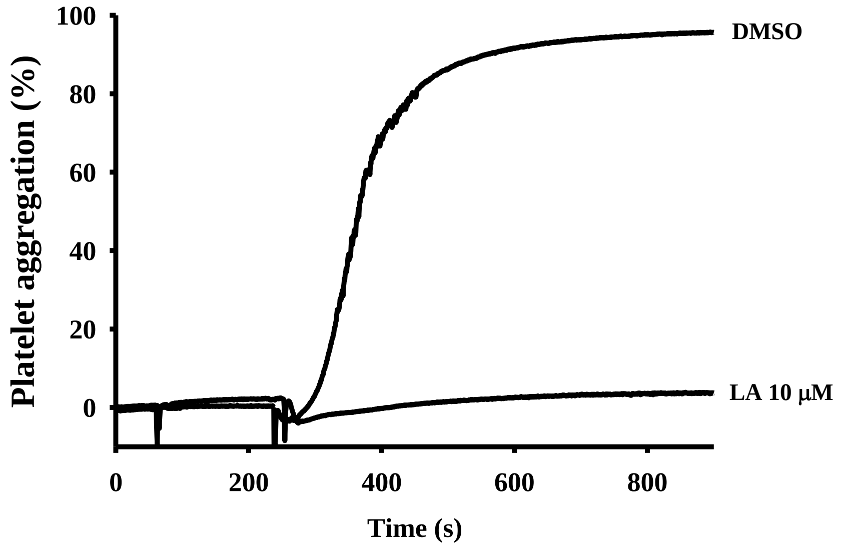

Figure S2. Effects of LA on AA-stimulated platelet aggregation in human platelets. Washed platelets were pre-incubated with LA (10 µM), followed by treatment with 60 µM AA to stimulate platelet aggregation. Profiles are representative of 3 similar experiments.

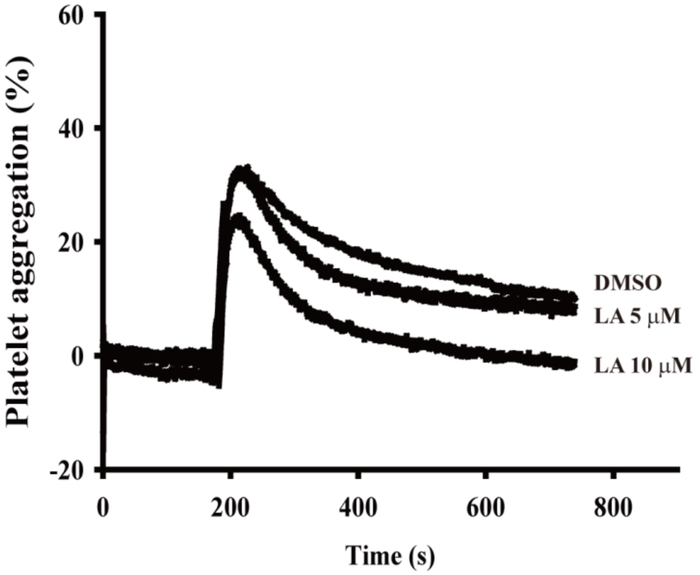

Figure S3. Effects of LA on ADP-stimulated platelet aggregation in mouse platelets. Mouse platelets were pre-incubated with LA (5 and 10 μM), followed by treatment with 5 μM ADP to stimulate platelet aggregation. Profiles are representative of 3 similar experiments.
